# The Effect of Kinesitherapy on Bone Mineral Density in Primary Osteoporosis: A Systematic Review and Meta-Analysis of Randomized Controlled Trial

**DOI:** 10.1155/2020/5074824

**Published:** 2020-08-05

**Authors:** Shanxi Wang, Shuzhen Li, Xing Xie, Juying Xie

**Affiliations:** ^1^College of Rehabilitation, Xiangnan University, Chenzhou 423000, China; ^2^Affiliated Hospital of Xiangnan University, Chenzhou 423000, China

## Abstract

**Objective:**

Osteoporosis (OP) is a well-established age-related disease, pathologically characterized by bone microarchitectural deterioration, increased fragility, and low BMD. Primary osteoporosis (POP) is the most common type of OP.

**Methods:**

Publications pertaining to the effectiveness of kinesitherapy on BMD in POP from PubMed, SCI, Cochrane Library, Embase, VIP, CNKI, and Wanfang Database were retrieved from their inception to October 2019.

**Results:**

A total of 21 studies with 1840 participants were included. The results of the meta-analysis revealed that kinesitherapy plus antiosteoporosis medications had a positive effect on lumbar spine BMD when the duration of intervention was 6 months (MD = 0.11 g/cm^2^; 95% CI: 0.06–0.15; *P* < 0.0001) or >6 months (MD = 0.04 g/cm^2^; 95% CI: 0.02–0.06; *P* < 0.0001) compared with antiosteoporosis medications alone. Additional kinesitherapy plus antiosteoporosis medications were associated with improved femoral neck BMD compared with antiosteoporosis medications alone (MD = 0.09 g/cm^2^; 95% CI: 0.03–0.16; *P*=0.004).

**Conclusions:**

Kinesitherapy plus antiosteoporosis medications significantly improved lumbar spine and femoral neck BMD in the current low-quality evidence. Additional high-quality evidence is required to confirm the effect of kinesitherapy on BMD in patients with POP.

## 1. Introduction

With age, bone mass declines, and an accelerated loss of bone mineral density (BMD) occurs by 50 years of age [1]. Osteoporosis (OP) is a well-established age-related disease pathologically characterized by bone microarchitectural deterioration, increased fragility, and low BMD [2]. The diagnosis of OP is based on measurements of BMD and is defined by the World Health Organization as BMD ≥ 2.5 standard deviations (SD) below the average value for young healthy individuals [3]. Moreover, OP is associated with high morbidity and mortality, as well as reduced quality of life, which in turn increases the rate of fractures, healthcare costs, and social economic stress [4–9]. In North America [10, 11], over 55 million people are at risk of developing OP or osteopenia. OP is typically classified into three main categories: (1) primary; (2) secondary; and (3) idiopathic. Primary osteoporosis (POP) refers to the natural aging process of human tissue and organ systems, and the symptoms are associated with degenerative changes in the skeletal system. Moreover, POP is the most common type of OP, accounting for 90% of OP cases, and includes women with postmenopausal osteoporosis (PMOP) and senile osteoporosis (SOP) [12, 13]. PMOP is primarily related to postmenopausal estrogen deficiency, whereas SOP is associated with increased age [14]. Thus, as the proportion of elderly populations increases throughout the world, the number of POP cases will also increase gradually. In the European Union, according to relevant statistics [15, 16], 22 million women and 5.5 million men over the age of 50 suffer from osteoporosis. And that number is expected to increase by 23 percent by 2025, according to a study. One study found that the overall prevalence rate of osteoporosis was 32.1% in at least one measurement site (28.5% in the lumbar and 14.5% in the femoral region), while 49.7% of elderly people suffer from decreased bone mass (osteopenia) in Amirkola, north of Iran [17]. According to the latest epidemiological results of osteoporosis in China, the prevalence of osteoporosis at the age of 40∼49 is 3.2%, including 2.2% in males and 4.3% in females. The prevalence rate of osteoporosis over the age of 50 was 19.2%, including 6.0% in males and 32.1% in females. And the prevalence of osteoporosis over the age of 65 was 2.0%, with 0.7% of men and 51.6% of women [18].

POP treatment is a long-term process and may not 100% prevent the development or reverse the symptoms of the disease [19]. In addition, exercise is one of the key recommendations for the prevention and treatment of bone loss [20, 21]. Several studies have demonstrated that exercise can prevent bone loss [22] and improve calcium absorption, bone formation [23], and the secretion of sex hormones [24, 25], which then promotes BMD [26]. Kinesitherapy, as a part of physical therapy, is a comprehensive exercise that represents one of the most important aspects of medical rehabilitation. Kinesiotherapy involves the movement of various parts of the body or the entire body using exercises to maintain, establish, develop, and change the function of the locomotor apparatus and organs in locomotion. Kinesiotherapy for the treatment of POP primarily includes routine static training, walking training, grip training, outbreak and endurance exercise training, push-ups, stretching, or isometric exercise. In addition, multiple reviews have confirmed that exercise reduces bone loss and increases BMD in postmenopausal women or PMOP [27–29]. A meta-analysis also demonstrated that exercise can improve functional outcomes, including mobility, balance, and self-reported measures of functioning in persons with OP [30]. However, there was no systematic review to evaluate the effect of kinesitherapy on BMD in patients with POP. Therefore, the aim of this study was to conduct a systematic review and meta-analysis to assess the effect of kinesitherapy in persons with POP on lumbar spine and femoral neck BMD via conducting a maximal search of both Chinese and English databases.

## 2. Methods

### 2.1. Eligibility Criteria

Available human, clinical, or community studies with a randomized controlled trial published in English or Chinese were included in this review. The participants consisted of patients with POP who had no thoracolumbar vertebral fracture and other complications such as heart, vein, lung, liver, and kidney as well as metabolic diseases and were not taking drugs affecting bone metabolism. The age and gender of the subjects were not limited. The included studies focused on the effect of kinesitherapy plus antiosteoporosis medications therapy as a kinesitherapy group compared with antiosteoporosis medications therapy as a control group for the BMD of POP (SOP and SMOP). Those which compared kinesitherapy alone with another exercise or any other antiosteoporosis intervention were excluded. The kinesitherapy should include weight-bearing, impact, resistance, endurance training, or a combination of these types of training, and only single-motion experiments will be ruled out. Health education can be added to all patients, and all inpatients can be given routine care. The outcomes included at least lumbar spine or femoral neck BMD.

### 2.2. Data Sources and Searches

The original research articles were obtained after a search of six electronic English and Chinese databases, which included PubMed, Science Citation Index (SCI), EMBASE, Chinese Scientific Journal Database (VIP), China National Knowledge Information (CNKI) database, and Wanfang from their inception to October 3, 2019. We used the following search strategy ([kinesitherapy OR exercise] AND osteoporosis AND bone mineral density) in all the English and Chinese databases.

### 2.3. Study Identification and Quality Assessment

Two reviewers (WSX and LSZ) independently screened and reviewed the title and abstract of the searched studies using NoteExpress V3.2.0.6992 software. The full text of the studies that potentially met the eligibility criteria was obtained, and any potentially relevant references were retrieved according to predefined eligibility criteria. The data were extracted by one reviewer (WSX) using the prepared forms and checked for accuracy by the second reviewer (LSZ). The details extracted from the eligible literature included the language of publication, participant characteristics, sample size, methodological information, participant demographics, experimental and control interventions (category, intensity, frequency, duration, and details of antiosteoporosis medication treatment), outcomes, and adverse effects [31]. Studies published in multiple reports were only included once to avoid duplication in this review. Disagreements were resolved through discussion, and the original author was contacted if an agreement could not be reached. The primary outcomes were lumbar spine BMD and femoral neck BMD, which were expressed as g/cm^2^ assessed by dual X-ray absorptiometry or dual photo absorptiometry. The baseline and follow-up data pertaining to BMD were calculated. If follow-up data could not be obtained, the data at the end of the intervention were used instead.

The quality of the studies was independently evaluated by two reviewers (WSX and LSZ) using the Cochrane Collaboration's tool for assessing the risk of bias [32]. The following recommended domains were considered: selection bias (random sequence generation and allocation concealment); performance bias (blinding of participants and personnel); detection bias (blinding of outcome assessment); attrition bias (incomplete outcome data); reporting bias (selective reporting); and other bias, each of which was rated according to the level of bias and categorized as either low, high, or unclear.

### 2.4. Data Analysis

Review Manager 5.2 software from the Cochrane Collaboration was applied for the data analysis. Statistical heterogeneity among the studies was assessed using a chi-square test or by calculating Higgins *I*^2^ values [33]. The results were pooled using a fixed effect model when the *I*^2^ value was less than 40%. Otherwise, a random effect model was applied. However, if the *I*^2^ value among the studies was greater than 75%, the heterogeneity was considered to be substantive and the overall meta-analysis was not appropriate to conduct, but a sensitivity analysis was considered to measure the pooled effect. Subgroup analysis was used to explore the source of heterogeneity if the duration of the intervention exhibited variability. The continuous outcomes were calculated for the mean difference (MD) with standard deviations (SDs) with a corresponding 95% confidence interval (CI), and all *P* values were two sided.

## 3. Results

### 3.1. Description of Studies

**Figure 1:**
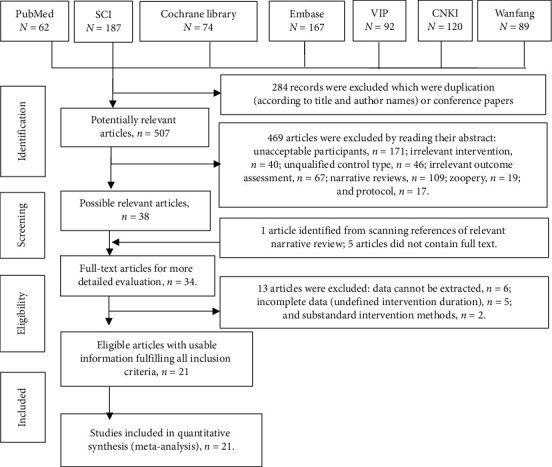
Details of literature search and selection. SCI: Science Citation Index; VIP: Chinese Scientific Journal Database; CNKI: China National Knowledge Information database.

A detailed screening flowchart depicting the generation of eligible articles is presented in Figure 1. A total of 791 records were identified via database searches. After removing duplicates, 507 remained to be screened for eligibility. Consequently, 21 studies met the inclusion criteria and were included in the meta-analysis.

Table 1 presents the sample, intervention, and outcome characteristics. This review involved a total of 1840 POP patients (including SOP and PMOP). Part of the subjects were from outpatient clinics, inpatient settings, and community or physical examination centers, with the exception of eleven subjects for whom the sources were unknown. In the included studies, the subject type consisted of eleven PMOP patients, five POP patients, and five SOP patients. In one RCT, the diagnostic criteria for the study were in accordance with the WHO diagnostic criteria for osteoporosis. In the seven RCTs, the reference diagnostic criteria for osteoporosis were Chinese, with one diagnostic criterion for osteoporosis in Japan; the Chinese diagnostic criteria included the Chinese recommended diagnostic criteria for osteoporosis, the Chinese medical association osteoporosis diagnosis and treatment guidelines (a second draft) and a primary bone guide formulated by Chinese Medical Association Osteoporosis and Bone Mineral Disease Branch; one RCT used Western diagnostic criteria for disease; four RCTs only mentioned the diagnostic criteria in accordance with the diagnostic criteria of PMOP or POP; and five RCTs used the laboratory examination in which the *T* value of was less than or equal to −2.5 SD in at least one site; the diagnostic criteria in the remaining RCT did not elaborate. The whole RCTs compared kinesitherapy plus medication treatment with medication treatment alone. Kinesitherapy involves comprehensive exercises rather than individual exercises. Of those RCTs, only one RCT used traditional Chinese medicine (e.g., kidney method), and the remaining RCTs were treated with Western medicine. All of the RCTs included a measure of lumbar spine BMD, and five of the RCTs included lumbar spine and femoral neck BMD.

### 3.2. Methodological Quality

**Figure 2:**
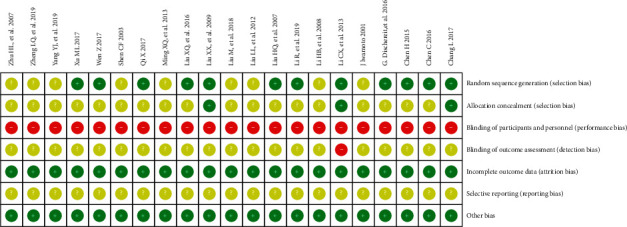
Risk of bias summary for each included study.

As shown in Figure 2, twelve studies described the generation of random sequences. Five of these studies used a random number table, three of these used simple random methods, three studies used computer-generated random numbers, and the remaining one trial used the method of lottery. Three trials involve allocation concealment. However, the blind intervention associated with the intervention exercises cannot be implemented blindly. One study described that the data analysis was based on the author's own statistics. One study described an exit from the case, but did not explain the reason. There were no dropouts indicated or explanations for withdrawal in the remaining studies. All of the included studies were considered to have a high risk of bias.

### 3.3. Meta-Analysis of Lumbar Spine BMD

All the controls in the literatures were kinesitherapy plus antiosteoporosis drugs versus antiosteoporosis drugs. According to the duration of the intervention, the subgroups were divided into three groups based on an intervention duration of (1) less than 6 months; (2) 6 months; and (3) longer than 6 months.

#### 3.3.1. Intervention Duration < 6 Months

**Figure 3:**
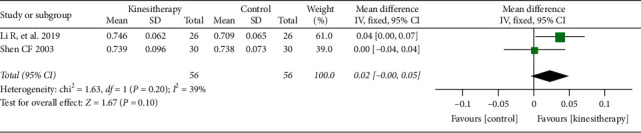
Kinesitherapy plus antiosteoporosis medications versus antiosteoporosis medications on lumbar spine BMD (intervention duration < 6 months).

Two trials [35, 53] compared the effect of kinesitherapy plus antiosteoporosis medications with antiosteoporosis medications alone on lumbar spine BMD when the duration of intervention was less than 6 months. The meta-analyses indicated that there was no significant difference between the two groups (MD = 0.02; 95% CI: −0.00–0.05; *P*=0.10) (Figure 3).

#### 3.3.2. Intervention Duration = 6 Months

**Figure 4:**
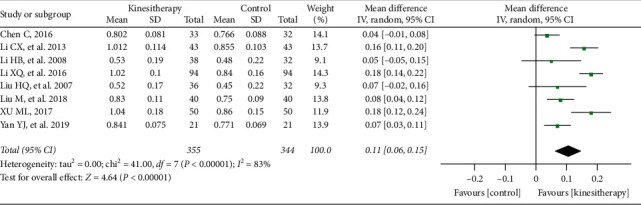
Kinesitherapy plus antiosteoporosis medications versus antiosteoporosis medications on lumbar spine BMD (intervention duration = 6 months).

Ten studies [37, 38, 41, 46, 51, 54] involving 699 participants reported that after 6 months, kinesitherapy had significantly increased lumbar BMD (MD = 0.11 g/cm^2^; 95% CI: 0.06–0.15; *P* < 0.0001). However, the heterogeneity among the studies was substantial with *I*^2^ = 83%, and no obvious changes were observed after the sensitivity analysis when any one or two of the studies were removed (Figure 4).

#### 3.3.3. Intervention Duration ＞ 6 Months

**Figure 5:**
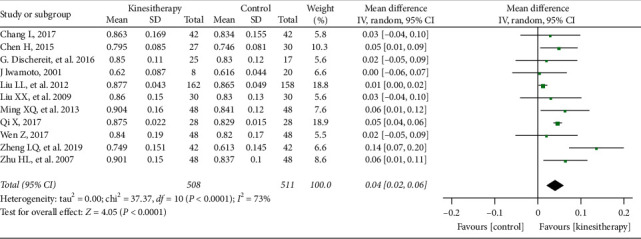
Kinesitherapy plus antiosteoporosis medications versus antiosteoporosis medications on lumbar spine BMD (intervention duration > 6 months).

Eleven studies [34, 36, 39, 40, 42–44, 48–50, 52] involving 1019 participants revealed that when the duration of treatment was longer than 6 months, the lumbar spine BMD in the kinesitherapy group significantly increased compared with the control group (MD = 0.04 g/cm^2^; 95% CI: 0.02–0.06; *P* < 0.0001) with high heterogeneity (*I*^2^ = 73%) (Figure 5). The heterogeneity was *I*^2^ = 23% after the sensitivity analysis when one study [40] was removed.

### 3.4. Meta-Analysis of Femoral Neck BMD

**Figure 6:**
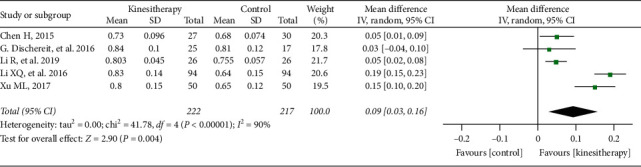
Meta-analysis of femoral neck BMD.

Five trials [43–45, 47, 53] involving 439 participants compared effect of kinesitherapy plus antiosteoporosis medications with antiosteoporosis medications alone on lumbar spine BMD. The meta-analysis revealed a significant antiosteoporosis effect on lumbar spine BMD (MD = 0.09 g/cm^2^; 95% CI: 0.03–0.16; *P*=0.004) but with high heterogeneity (*I*^2^ = 90%) (Figure 6). It showed low heterogeneity (*I*^2^ = 0%) after the sensitivity analysis when two studies were removed [45, 47].

## 4. Discussion

### 4.1. Summary

POP is a worldwide health problem that primarily impacts postmenopausal women and senile individuals. Moreover, POP is often related to physical frailty, an increased risk of falls, substantial morbidity, mortality, and impairment in quality of life [55]. The aim of OP treatment is to improve BMD and prevent fractures. Nonpharmacological treatment includes a healthy diet, prevention of falls, and physical exercise programs. Pharmacological treatment involves calcium, vitamin D, and medications for activating bone tissue (e.g., antiresorptives, bone formers, and mixed agents) [56]. In addition, exercise is considered important for maintaining bone health. Individuals with OP are strongly recommended to regularly engage in multicomponent exercise programs [57]. Moreover, several studies have confirmed that exercise can increase BMD at the femoral neck and the lumbar spine in elderly women with osteoporosis [58, 59]. This review is the first systematic review and meta-analysis to evaluate the effect of kinesitherapy on BMD on the lumbar spine and femoral neck in persons with POP from RCTs. This study involved 21 RCTs that included a total of 1840 subjects with POP (including SOP and PMOP). The duration of treatment varied from 3 to 24 months. The outcome measures primarily consisted of lumbar spine and femoral neck BMD. The results of the meta-analysis showed that there were no statistically significant differences between kinesitherapy plus antiosteoporosis medications versus antiosteoporosis medications alone on lumbar spine BMD when the duration of intervention was less than 6 months (MD = 0.02; 95% CI: −0.00–0.05; *P*=0.10). However, kinesitherapy had a positive effect on lumbar spine BMD when the duration of intervention was 6 months (MD = 0.11 g/cm^2^; 95% CI: 0.06–0.16; *P* < 0.0001) or more than 6 months (MD = 0.04 g/cm^2^; 95% CI: 0.02–0.06; *P* < 0.0001) compared with antiosteoporosis medications alone. Furthermore, kinesitherapy had a remarkable effect on femoral neck BMD (MD = 0.09 g/cm^2^; 95% CI: 0.03–0.16; *P* < 0.004) when compared with antiosteoporosis medications alone.

### 4.2. Limitations and Suggestions for Future Research

A total of 21 RCTs were included in this review, which showed that kinesitherapy had a favourable effect on lumbar spine and femoral neck BMD in patients with POP. Nevertheless, the interpretation and generalization of this systematic review and meta-analysis are subject to some limitations. According to the Cochrane Collaboration's tool, low-quality evidence, which included studies with a high risk of bias, resulted in a high heterogeneity of the meta-analysis results and favoured the positive effect of kinesitherapy on BMD in patients with POP. There were eleven RCTs that did not report the random sequence generation, and the remaining RCTs were lacking detailed descriptions of randomization, which could result in selection bias. The performance bias was high since the blinding of participants and personnel was not implemented. In all of the included trials, whether the blinding of the outcome assessment was used or not is not mentioned except for one study which states explicitly that the blinding of the outcome assessment was not applied and the statistics of outcome operated by the author. Although one trial reported reasons for withdrawal and dropout, an intention-to-treat analysis was not performed in the data analysis phase for which attrition bias was inevitable. In addition, all of the study protocols from the trials could not be obtained. Furthermore, a specific exercise was not designed for the analysis, which suggests that such an analysis is problematic due to the diversity of interventions. Only five trials included the outcome of femoral neck BMD in the currently available studies; thus, the reliability of the treatment effects of kinesitherapy on femoral neck BMD is reduced. Therefore, more multicenter, larger sample, long-term, single-blind RCTs are required to assess the effect of kinesitherapy on BMD in patients with POP.

## 5. Conclusion

The meta-analyses in this study suggest that kinesitherapy plus antiosteoporosis medications can significantly improve lumbar spine BMD when the duration of intervention is longer than 6 months compared with antiosteoporosis medications alone in the current low-quality evidence. More high-quality evidence in the form of multicenter, larger sample, long-term, single-blind, randomized controlled trials is required to confirm the effect of kinesitherapy on BMD in patients with POP.

## Figures and Tables

**Table 1:** The characteristics of all the trails.

Author, year	Participants (type, source, age, sample)	Duration (months)	Intervention		Outcomes
			Kinesitherapy group	Control group	
Iwamoto et al., 2001 [34]	PMOP, unspecified, 53–77 years, 28 (KT: 8, CON: 20)	24	Brisk walking (1000 steps in the first 7 days, increase the step count by 30%/week) + gymnastic training (no details provided)) + CON	Calcium lactate (2.0 g, Qd) and 1*α*-hydroxyvitamin D3 (1 *µ*g, Qd)	Lumbar spine BMD

Shen, 2003 [35]	POP, outpatient and inpatient, 45–80 years, 60 (KT: 30, CON: 30)	3	Aerobics, tai chi, dance, yangko, jogging, walking etc. (30–60 min/time, 5–7 times/week) + CON	Tonifying kidney granules (3 times/day, 1 dose/time)	Lumbar spine BMD

Zhu, 2007 [36]	SOP, outpatient, 60–72 years, 96 (KT: 48, CON: 48)	12	Walking, jogging, tai chi (30–60 min/time, 3–5 times/week) + CON	Calcium (600 mg/d)	Lumbar spine BMD

Liu et al., 2007 [37]	PMOP, outpatient, 48–61 years, 68 (KT: 36, CON: 32)	6	Draft movement, abdominal isometric exercises, flexion, and extension of the upper limbs (20 minutes each time, once every 3 days) + CON	Caltrate D (600 mg, Qd)	Lumbar spine BMD

Li et al., 2008 [38]	PMOP, unspecified, 48–61 years, 70 (KT: 38, CON: 32)	6	Draft movement, abdominal isometric exercises, flexion, and extension of the upper limbs (20 min/time, once every four days) + CON	Ossotide injection (50 mg, Qd, 20 days in total)	Lumbar spine BMD

Liu et al., 2009 [39]	SOP, outpatient service, 60–94 years, 60 (KT: 30, CON: 30)	12	Walking, jogging, tai chi (60 min/time, 1 time/day) + CON	Fosamax (10 mg, once a day) and calcium (600 mg/d)	Lumbar spine BMD
Liu and Wang, 2012 [40]	SOP, unspecified, 60–81 years, 320 (KT: 162, CON: 158)	12	Tai chi and jogging (no less than 30 min/time, no less than 4 times/week) + CON	Calcium carbonate D_3_ (600 mg, Qd)	Lumbar spine BMD
Li et al., 2013 [41]	SOP, hospital, 67 ± 4 years, 86 (KT:43, CON: 43)	6	Progressive lumbar dorsal muscle function exercise includes sitting training, swallow training and five-point support training (1-2 times/day) + CON	Lorelli calcium capsule (l capsules, 1 time/d for 2 consecutive months)	Lumbar spine BMD

Ming, 2013 [42]	SOP, hospital, 60–78 years, 96 (KT: 48, CON: 48)	12	Walking, aerobics, running ,and tai chi (5 to 7 times a week for 45 to 60 minutes)+ CON	Calcium gluconate (20 ml/time, 3 times/day) and vitamin D (400 units, 2 times/day)	Lumbar spine BMD

Chen, 2015 [43]	PMOP, clinic, 53– years, 57 (KT: 27, CON: 30)	12	Brisk walking (15–30 minutes), resistance strength exercises (15–20 minutes), and balance and flexibility exercises (simplify tai chi and five birds, 15–20 min) + CON	Alendronate (70 mg, Qd), caltrate D (600 mg, Qd), and alfacalcidol soft capsules (0.25 *μ*g, Qd)	Lumbar spine and femoral neck BMD

Dischereit et al., 2016 [44]	PMOP, unspecified, 68 years, 42(KT: 25, CON: 17)	24	Endurance and strength training program (3 sessions once weekly, 65 min) + CON	Adequate calcium and vitamin D supplementation and bisphosphonate therapy	Lumbar spine and femoral neck BMD

Li et al., 2016 [45]	PMOP, unspecified, 52–76 years, 188 (KT: 94, CON: 94)	6	Mainly includes walking, aerobics, running, and tai chi (30–60 min/time, more than 3 times/week) + CON	Caltrate D (1000 mg, Qd), derivatives, vitamin D, and raloxifene (1 pill, Qd)	Lumbar spine and femoral neck BMD

Chen, 2016 [46]	PMOP, unspecified, 53–77 years, 65(KT:33, CON:32)	6	Brisk walking and tai chi (30–50 min/time, 2-3 times/week) + CON	Alendronate (70 mg,Qd), caltrate D (600 mg, Qd), and alfacalcidol soft capsules(0.25 *μ*g, Qd)	Lumbar spine BMD

Xu, 2017 [47]	PMOP, unspecified, 51–67 years, 100 (KT:50, CON:50)	6	Aerobics, tai chi, and jogging (more than 30 min, more than 3 times/week) + CON	Calcine D (2 times/day, 2pills/time) + estrogen (1 time/day, 60 mg/time)	Lumbar spine and femoral neck BMD

Chang, 2017 [48]	POP, unspecified, 60–79 years, 84 (KT: 42, CON: 42)	12	Aerobics, walking, swimming, jogging, and cycling (3–4 times, not less than 2 times, each exercise 30–60 minutes) + CON	Calcine D 600 (1 tablet once, 2 times a day) and health education	Lumbar spine BMD

Qi, 2017 [49]	PMOP, community healthcare center, 45–65 years, 56 (KT:28, CON:28)	12	Aerobic exercise resistance group, load bearing, and stretching (30 min/time, 3–5 times/week, more than 1 h) + CON	Conventional treatment	Lumbar spine BMD

Wen, 2017 [50]	POP, unspecified, 60–78 years, 96 (KT: 48, CON: 48)	12	Jogging, tai chi, etc. (daily) + CON	Routine prevention and taking medicine	Lumbar spine BMD

Liu and Yang, 2018 [51]	POP, unspecified, 36–79 years, 80 (KT: 40, CON: 40)	6	Walking, fitness running, ballroom dancing, and swimming (at least 12 times a month, each time ≥30 min) + CON	Calcium and vitamin D3	Lumbar spine BMD

Zheng et al., 2019 [52]	POP, unspecified, 53–77 years, 84 (KT: 42, CON: 42)	12	Walking, jogging, alternate running, cycling, etc. (3 to 4 times per week, the minimum 2 times, 30–60 min) + CON	Calcium (300 mg/tablet, 2 times/d,1 tablet/time)	Lumbar spine BMD

Li et al., 2019 [53]	PMOP, unspecified, 50–65 years, 52 (KT: 26, CON: 26)	3	Brisk walking (30 min, once a day, 4d/week) and resistance training (week 1, 2 weekly complete 1 set (15 times/set), and then add 1 set every 2 weeks) + CON	Calcium carbonate D3 (600 mg, 1 time/d), alfacalcidol soft capsule (0.5 g, 1time/d), and sodium alendronate (70 mg, 1 time/d/weeks) for 3 months	Lumbar spine and femoral neck BMD

Yan et al., 2019 [54]	POP, unspecified, 53–77 years, 52 (KT: 26, CON: 26)	6	Flexible resistance exercise therapy to exercise the lumbar and dorsal muscles (5 times/week) + CON	Calcium carbonate D3 (600 mg), vitamin D3 (0.25 UG), health education, and routine nursing	Lumbar spine BMD

PMOP, postmenopausal osteoporosis; POP, primary osteoporosis; SOP, senile osteoporosis; KT, kinesitherapy group; CON, control group; BMD, bone mineral density.
